# 599 Age-Related Differences in Global Burn Outcomes: Comparative Analysis of Pediatrics, Young Adults, Adults, and Geriatrics

**DOI:** 10.1093/jbcr/iraf019.228

**Published:** 2025-04-01

**Authors:** Daniel Najafali, Hilary Liu, Megan Najafali, José Arellano, Saeid Rezaei, Logan Galbraith, Mare Kaulakis, Erik Reiche, Francesco Egro

**Affiliations:** Carle Illinois College of Medicine; University of Pittsburgh Medical Center; Loyola University Chicago Stritch School of Medicine; University of Pittsburgh Medical Center; University of Pittsburgh Medical Center; Northeast Ohio Medical University; University of Pittsburgh School of Medicine; University of Pittsburgh Medical Center; University of Pittsburgh Medical Center

## Abstract

**Introduction:**

Burn outcomes vary significantly across different age groups. There is limited data on how age influences burn outcomes in low- and middle-income countries (LMIC) where resources may be limited. Many studies on age isolate pediatric or adult groups while focusing on datasets from high-income regions. This study aims to evaluate the impact of age on burn injury outcomes in low- and high-resource areas. It was hypothesized that younger age would be protective with respect to burn injuries mirroring findings in high-income countries.

**Methods:**

Data from the Global Burn Registry was used to stratify individuals with burn injuries across cohorts based on their age: Pediatrics (0-17 years), Young Adults (18-39 years), Adults (40-64 years), and Geriatrics (65 years and older). Descriptive statistics were used to provide an overview of burn characteristics. Multivariable logistic regression analysis assessed if age was a predictor for the primary outcome of mortality and secondary outcome measures of surgical intervention and functional impairment at discharge for survivors.

**Results:**

The cohort (N=9,274) consisted of 3,757 pediatric cases, 3,108 young adults, 1,798 adults, and 611 geriatric patients. Pediatric patients comprised 41% of the sample, young adults 34%, adults 19%, and geriatrics 7%. Mortality rates increased significantly with increasing age, with pediatric patients having the lowest mortality (9%), followed by young adults (25%) and adults (25%), then geriatric patients (30%) (P< 0.001). Surgical rates were highest among adults (56%) and lowest among pediatrics (48%). Multivariable logistic regressions demonstrated that pediatric patients had significantly decreased odds of mortality (OR 0.65, 95%CI 0.51-0.81), lower odds of surgical intervention (OR 0.85, 95%CI 0.74-0.96), and lower odds of impairment at discharge for survivors (OR 0.72, 95%CI 0.57-0.91).

**Conclusions:**

Age is a critical determinant of burn injury outcomes on a global scale, with older age groups having significantly worse outcomes and impairment at discharge. Geriatric patients represent a particularly vulnerable patient population and would benefit from age-specific interventions such as increased monitoring and rehabilitation efforts.

**Applicability of Research to Practice:**

Optimizing burn outcomes can be achieved by understanding the differences that exist in outcomes across age groups. Younger age serves as a protective factor, whereas older age indicates a potentially worse prognosis.

**Funding for the Study:**

N/A

